# Using Machine Learning to Improve Screening for Oropharyngeal Dysphagia in Hospitalized Versus Primary Care Adult Patients With COVID-19 Disease: Retrospective Observational Study

**DOI:** 10.2196/81028

**Published:** 2026-04-13

**Authors:** Cristina Amadó Ruiz, Alberto Martín, Jaume Miró Ramos, Francisco Javier Ruz Torres, Antonio Ruiz, Adil El Haji, Pere Clavé, Omar Ortega

**Affiliations:** 1 Gastrointestinal Physiology Laboratory, Department of Surgery, Hospital Universitari de Mataró, Consorci Sanitari del Maresme, Universitat Autònoma de Barcelona, C. Cirera s/n, Barcelona, 08304, Spain, 34 937417700; 2 Artificial Intelligence Massive Screening—Medical SL (AIMS MEDICAL), Mataró, Spain; 3 Centro de Investigación Biomédica en Red de Enfermedades Hepáticas y Digestivas (CIBERehd), Instituto de Salud Carlos III, Madrid, Spain; 4 Fundació Salut Consorci Sanitari del Maresme, Mataró, Spain; 5 IT Department, Hospital de Mataró (Universitat Autònoma de Barcelona), Maresme Health Consortium, Mataró, Spain

**Keywords:** oropharyngeal dysphagia, swallowing disorders, COVID-19, machine learning, AIMS-OD, *ICD-10* coding

## Abstract

**Background:**

Oropharyngeal dysphagia (OD) commonly occurs in patients with COVID-19 disease, posing diagnostic challenges due to isolation protocols.

**Objective:**

This study aimed at evaluating Artificial Intelligence Massive Screening for Oropharyngeal Dysphagia (AIMS-OD), a machine learning software for real-time OD screening, comparing OD prevalence and clinical outcomes using OD *ICD-10* (*International Statistical Classification of Diseases, Tenth Revision*) R13 codes (R13-OD) and high-risk AIMS-OD (H-AIMS-OD) scores (>0.5), in hospital and primary care patients with COVID-19 disease. It explored clinical characteristics, OD risk factors, and clinical outcomes.

**Methods:**

This retrospective, observational study analyzed patients with SARS-CoV-2 aged 18 years and older in Catalonia from January 1 to August 31, 2020, including hospital and primary care data on clinical information, *International Classification of Diseases, Tenth Revision* (*ICD-10*) codes, hospital stay, discharge destination, and mortality. AIMS-OD assessed OD risk, stratifying patients by age (aged 18‐69 years and 70 years and older).

**Results:**

Among 257,541 patients with COVID-19 disease, 59.3% (152,721/257,541) were aged 18‐69 years and 40.7% (104,820/257,541) were aged 70 years and older. Hospital and primary care R13-OD prevalence was 3.5% and 4.3%, respectively; AIMS-OD showed 34.8% and 15.4%, with True prevalence at 16.7% and 7.4%. Patients aged 70 years and older had worse clinical outcomes and worse prognosis. Patients in R13-OD experienced significantly worse clinical outcomes than patients with H-AIMS-OD, who in turn fared worse than those with no R13-OD and with low AIMS-OD risk. Risk factors for patients with COVID-19 R13-OD included age, neuroleptic use, stroke, dementia, and delirium.

**Conclusions:**

AIMS-OD screening revealed high prevalence and significant underdiagnosis in patients with COVID-19 disease across settings. Early detection and risk stratification using AIMS-OD could improve clinical decision-making, diagnosis, and management, particularly in older patients with comorbidities.

## Introduction

COVID-19 pandemic originated in the city of Wuhan, China, in late 2019. It is a serious illness caused by SARS-CoV-2. The infection spread globally and was declared a pandemic by the World Health Organization on March 11, 2020 [1], and the ﬁrst case in Catalonia, Spain, was reported on February 25, 2020. The COVID-19 pandemic had devastating effects worldwide, with high rates of severely ill patients and mortality [2]. The most common symptoms of the disease were fever, cough, difficulty breathing, fatigue, loss of taste or smell, sore throat, and diarrhea [3].

Mortality from the COVID-19 pandemic in older patients was very high during the initial waves of the pandemic. This vulnerability was linked to the biological wear and tear inherent to age, to the higher prevalence of comorbidities, and to the fact that up to 50% of older patients requiring hospitalization were malnourished [4]. Several factors associated with increased disease severity and poor prognosis in COVID-19 have been identified, including advanced age, oropharyngeal dysphagia (OD), malnutrition (MN), frailty, and impaired functionality [5], as well as chronic conditions such as type 2 diabetes mellitus, arterial hypertension, or obesity and diseases of the immune system [4].

The first wave of the pandemic in Spain occurred from the second half of March to the second half of June 2020. During that period, we found that of the 205 patients with COVID-19 disease admitted consecutively to the Hospital de Mataró, 51.7 % (106/205) had OD on admission and 43.8% (90/205) had OD on discharge [5]. OD is a symptom of a swallowing disorder that is characterized by the inability to safely and effectively move the alimentary bolus from the mouth to the esophagus [6]. This condition has been recognized as a geriatric syndrome [7], acknowledged by the World Health Organization [8], and classified in the ICD (*International Classification of Diseases*) with codes 787.2 *ICD-9* (*International Classification of Diseases, Ninth Revision*), R13 *ICD-10* (*International Statistical Classification of Diseases, Tenth Revision*), and MD93 *ICD-11* (*International Classification of Diseases, 11th Revision*). OD is highly prevalent among different phenotypes of patients [6,9], including those with COVID-19 disease [5,10]. Moreover, OD is associated with severe complications, including MN, dehydration (DH), respiratory infections, aspiration pneumonia (AP), reduced quality of life, and increased mortality [8,11]. Recent prospective studies have further documented the high prevalence of OD in populations with COVID-19 disease using bedside screening methods. Zayed et al [12] reported that 45.4% of patients hospitalized with COVID-19 disease tested positive for OD using the Eating Assessment Tool and the Yale Swallowing Protocol, with risk factors including advanced age, dysphonia, ageusia, anosmia, intensive care unit (ICU) admission, and mechanical ventilation. In a subsequent study using instrumental assessment on patients with OD after COVID-19 disease, the same authors identified swallowing abnormalities using fiberoptic endoscopic evaluation of swallowing (FEES), including delayed activation of the swallowing reflex and altered laryngeal sensitivity, reinforcing the clinical relevance of systematic screening for OD in this population [13].

A swallow screening test is defined according to Swigert et al [14] as a minimally invasive evaluation procedure that quickly “determines the likelihood that dysphagia exists; whether the patient requires referral for further swallowing assessment; whether it is safe to feed the patient orally (for the purposes of nutrition, hydration, and administration of medication); whether the patient requires referral for nutritional or hydrational support.”

It is essential for health care centers to implement a systematic screening process for OD in patients with COVID-19 disease to identify those who require clinical evaluation for accurate diagnosis and appropriate compensatory treatment to avoid respiratory and nutritional complications. The diagnostic algorithm for OD involves a 3-step approach: screening, clinical assessment, and instrumental evaluation. The screening incorporates a specific anamnesis and validated questionnaires to identify OD risk factors [15]. Failure in screening leads to reduced diagnosis rates, increasing clinical risks, and health care costs [15,16]. If a patient is positive, a subsequent clinical assessment has to be performed involving the evaluation of clinical signs of impaired efficacy and safety of swallow to be able to prescribe the first compensatory treatment for the patient. The Volume-Viscosity Swallow Test is a clinical method with high sensitivity (93.17%) and specificity (81.39%) for OD diagnosis that uses different volumes and viscosities to clinically assess deglutition [15,17,18]. Finally, the instrumental assessment uses gold standard techniques such as videofluoroscopy and FEES, enabling the objective evaluation of deglutition mechanisms and the understanding of the pathophysiology of OD, including aspiration mechanisms [19,20].

Nowadays, there are automatic screening tools that have been developed using artificial intelligence (AI). AI is a branch of computer science capable of analyzing complex medical data. Its potential to explore meaningful relationships within a dataset can be used in the diagnosis, treatment, and outcome prediction in many clinical scenarios [21]. Furthermore, machine learning (ML) algorithms used in AI have demonstrated their potential in offering solutions to intricate issues [22]. Recent work in the field of AI applied to health care emphasizes that, beyond predictive performance, AI-based tools intended for clinical use must address issues related to interpretability, transparency, and clinical usability. In this regard, the ability of AI systems to rely on clinically meaningful variables and to support, rather than replace, clinical reasoning has been identified as a key factor for successful implementation in real-world settings. Moreover, current evidence highlights the expanding role of AI in health care not only for individual risk assessment but also for large-scale screening, population-level risk stratification, and improvements in health system efficiency, provided that these tools can be effectively integrated into existing clinical workflows and electronic health records (EHRs). In this context, AI-based screening approaches may offer complementary value by enabling systematic and scalable identification of patients at risk in scenarios where conventional clinical assessment is limited or not routinely feasible [23,24].

Artificial Intelligence Massive Screening for Oropharyngeal Dysphagia (AIMS-OD) [15] is a ML-based screening tool that has been developed to measure the risk of OD in acute hospitals, rehabilitation centers, nursing homes, and primary care. Specifically, AIMS-OD uses a nonlinear model built with Random Forest to predict the risk of OD in patients. The tool uses nonidentifying data, such as age, gender, Barthel index, and *ICD* codes [15].

The aims of this study are to demonstrate the underdiagnosis of OD in patients with COVID-19 across both hospitalized and primary care settings; to compare the prevalence of OD in patients with COVID-19 disease according to *ICD-10* coding (R13), group called R13-OD, or high-risk AIMS-OD scores (>0.5), group called H-AIMS-OD, in hospitalization patients and primary care settings; and to study the clinical characteristics, risk factors for OD, and clinical outcomes associated with 2 age groups: patients aged 18‐69 years and those aged 70 years.

## Methods

### Study Design

A retrospective observational and comparative study was conducted in a cohort that included all SARS-CoV-2-positive patients in Catalonia during the first wave of the COVID-19 pandemic. Prevalence of OD identified by the primary care and hospital clinicians and codified by *ICD* codes was compared with that determined by AIMS-OD (risk >0.5).

### Ethical Considerations

The study protocol was approved by the ethics committee of the Consorci Sanitari del Maresme (code: CEIm 43/20) and was conducted according to the principles and rules laid down in the Declaration of Helsinki and its amendments. Exemption of the informed consent form was granted by the institutional review board and followed the Guidance on the Management of Clinical Trials during the COVID-19 pandemic (European Commission, version 4; February 4, 2021). To ensure privacy and confidentiality, all data were anonymized prior to analysis and handled in accordance with the General Data Protection Regulation and applicable national regulations. No directly identifiable personal information was accessible to the research team. Participants did not receive any financial compensation for participation in this study.

### Study Population and Dataset

The study population included all patients aged 18 years and older who tested positive for SARS-CoV-2 (reverse transcription polymerase chain reaction with GeneXpert Dx [Cepheid]) in Catalonia from a database that included all Catalan patients from January 1 to August 31, 2020, at 2 levels of care, primary and hospital care. The first confirmed positive SARS-CoV-2 patient was reported in our database on February 26, 2020. Patient data were provided by the *Agència de Qualitat i Avaluació Sanitàries de Catalunya* [25]*, within the framework of the Analytical Program of Dades per a la Recerca i la Innovació en Salut* (PADRIS) [26]. The PADRIS program allows access to the reuse and cross-referencing of health data generated by the Sistema Sanitari Integral d’Utilització Pública de Catalunya [27] to promote research in the health field. PADRIS emphasizes the use of anonymized and deidentified data, which is a positive aspect in terms of privacy protection [26].

**Figure 1.**
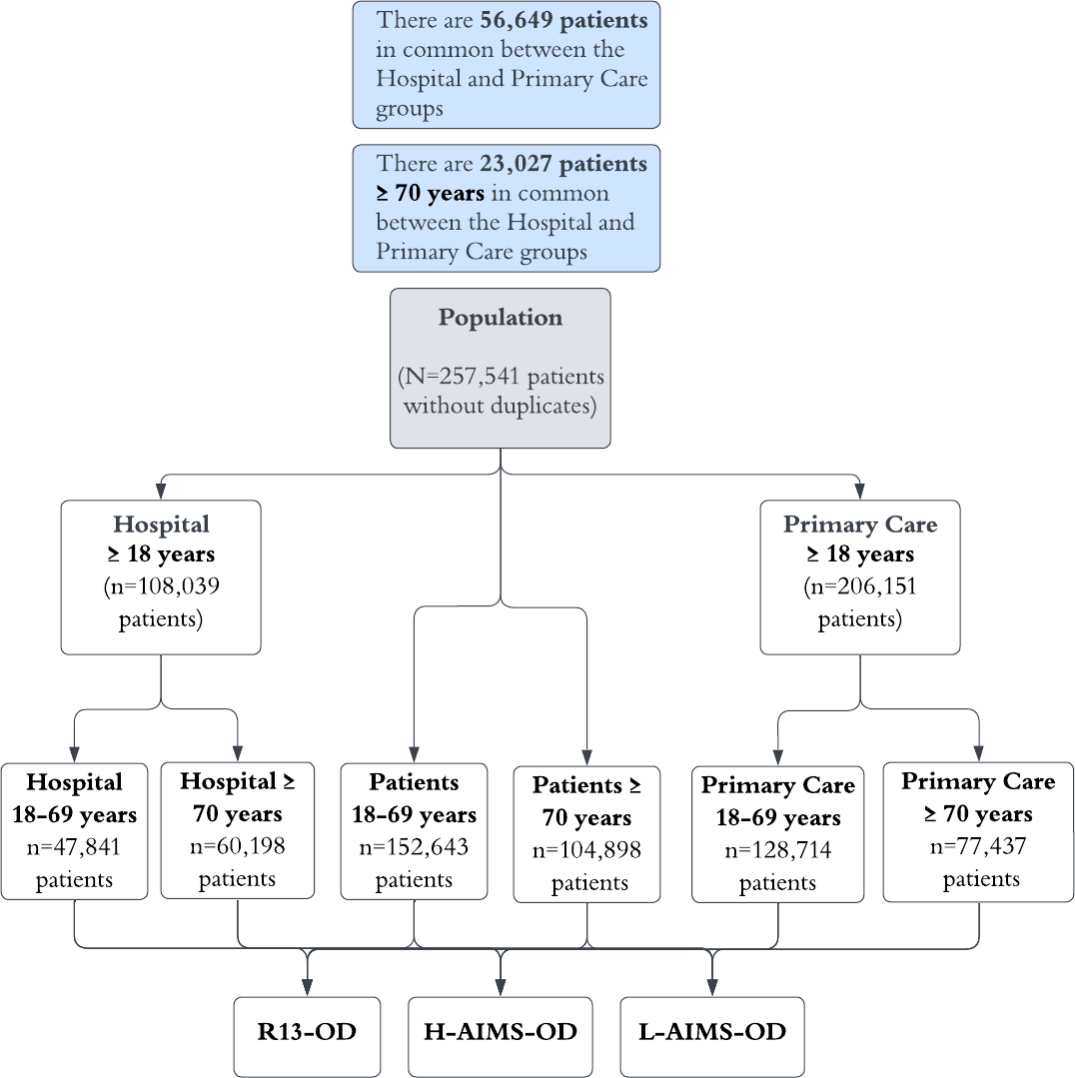
CONSORT (Consolidated Standards of Reporting Trials) flowchart showing the distribution of the whole study population into groups by setting and age range. H-AIMS-OD: high-risk Artificial Intelligence Massive Screening for Oropharyngeal Dysphagia; L-AIMS-OD: low Artificial Intelligence Massive Screening for Oropharyngeal Dysphagia; R13-OD: OD in patients with COVID-19 according to ICD-10 coding (R13).

The dataset was composed of 707,369 assistance records of the COVID-19 positive population, consisting of 257,541 patients who were admitted to hospital and/or treated in primary care centers. As illustrated in Figure 1, for the entire datasets (hospital and primary care), individuals were stratified into 2 categories: those aged between 18 and 69 years, and those aged 70 years and older. This initial division yielded 6 main groups, each of which was subsequently subcategorized into three distinct subgroups: (1) R13-OD, (2) H-AIMS-OD (sensitivity 0.950, specificity 0.304) [15] and, (3) those with no R13-OD and with no high risk of AIMS-OD, a group called with no R13-OD and low AIMS-OD risk (L-AIMS-OD). The *ICD-10* R13-OD diagnostic code was assigned during routine clinical practice by physicians based on clinical judgment and documentation, and these codes were present in the database of the study (retrospective data). The AIMS-OD risk categories (high risk or AIMS-OD risk of >0.5 vs low risk or AIMS-OD risk of <0.5) were generated retrospectively and automatically by the AIMS-OD algorithm using the anonymized health records data present in the study database. No speech-language pathologist, nurse, or physician manually classified patients into AIMS-OD risk groups for the purposes of this study.

### Clinical Variables and Measures

To establish a clinically consistent basis for comparison, the *ICD-10* code R13 for OD was used as the reference standard. Table 1 shows a summary of the study variables. The anatomical therapeutic chemical codes of drugs related to OD and the *ICD-10* codes during the previous 2 years and during the period from January 1 to August 31, 2020, were included.

**Table 1.** Variables of interest extracted through database processing, storage, and treatment.

Data type	Description	Variables, n
Demographic data	Sex and age	2
Diagnosis codes	*ICD-10*^a^ codes	1215
Dysphagia	Dysphagia (R13.0 to R13.19)	20
Clinical outcomes	AP^b^ (J69.0, J69.8), MN^c^ (E45, E44, E43), DH^d^ (E86), total mortality, 1-month mortality, 6-month mortality, and 1-year mortality	10
Administrative data	Length of hospital stay, discharge to nursing home, Charlson, admission to ICU^e^ and days in ICU	5
Anthropometric variables	Weight and BMI	2
Dispensed medication	Drugs related to OD^f^	20

a *ICD-10*: *International Classification of Diseases, Tenth Revision*.

b AP: aspiration pneumonia.

c MN: malnutrition.

d DH: dehydration.

e ICU: intensive care unit.

f OD: oropharyngeal dysphagia.

### Data Processing

Duplicated patients were removed from the database. Data manipulation methods were also used, such as the creation and deletion of aggregate variables, dummies, and variable collapse [28], to select the most relevant variables for our results.

In order to obtain the study variables, new variables derived from existing data were generated to determine the discharge to nursing homes, the number of days of hospital stay, and the number of readmissions for each patient. These variables provided complementary information on patient clinical status and outcomes.

For the variables age, weight, BMI, days of admission to the ICU and hospital stays, and number of admissions, the most recent record over time was selected as it is necessary to choose a single value for each variable. The processing of the database variables was performed with the programming languages R (R Project for Statistical Computing version 4.3.0; R Foundation for Statistical Computing) and Python (Python version 3.10.0; Python Software Foundation).

### ML Screening Software: AIMS-OD

The risk estimation service is offered through an application programming interface (API), allowing any facility with EHRs to request their patients’ OD risk. AIMS-OD has an area under the receiver operating characteristic curve of 0.840, sensitivity 0.940, specificity 0.416, positive predictive value 0.834, negative predictive value 0.690, positive likelihood ratio 1.61, and negative likelihood ratio 0.146 [15]. The web service process involves the following steps: (1) First, the health care facility anonymizes the clinical data of its EHR and sends a query via Hypertext Transfer Protocol Secure using the JavaScript Object Notation format, which is an international standard. (2) The API receives the query and verifies the user’s authorization and whether it contains the required fields and security measures. (3) Finally, the API sends the query to our expert system (ES), which predicts the risk of OD. The risk is returned as a number between 0 and 1 to the consulting health care facility. The ES system uses a proprietary algorithm that uses nonlinear ML methods to perform OD risk prediction. Both the ES and the API have been developed using open source software and are highly scalable as the system includes several API-based microservices [15].

To develop our predictive model, we drew on a comprehensive database created based on the knowledge of health care experts, established literature, and our team’s extensive knowledge of OD in older people. This expert database covers more than 25,000 potential variables, directly or indirectly related to OD. These variables covered the presence or absence of pathologies documented in the patients’ EHR in the 24 months prior to admission [15].

### Data Analysis and Statistical Methods

The prevalence and risk of OD; the prevalence of AP, MN, and DH; length of stay; discharge to nursing home; ICU admission; and the total mortality rate were the main outcome study variables. Several analyses were carried out: an initial descriptive statistical analysis with the entire group of patients described in the study design section and illustrated in Figure 1; a subsequent comparative analysis between the 3 groups (patients with COVID-19 disease according to R13-OD, H-AIMS-OD, and L-AIMS-OD groups); a comparative analysis between the proportions of OD prevalence in the overall, hospital, and primary care groups; and the true prevalence of OD in different age groups. We then performed descriptive and comparative analysis with the hospital and primary care groups (Figure 1), and finally bivariate and multivariate analysis for R13-OD and H-AIMS-OD.

For the comparative analysis between proportions, the prevalence of OD in patients with an R13-OD, the prevalence of OD in H-AIMS-OD patients, and the true prevalence of OD in patients in different age ranges were studied with the test of proportions based on the normality test (z). The estimated and true prevalences of the overall, hospital, and primary care patients were studied (Figure 1).

When estimating disease prevalence using imperfect screening tools (ie, those with sensitivity and specificity less than 100%), the observed prevalence is subject to misclassification bias. To obtain an unbiased estimate of the “True prevalence,” a standard epidemiological correction method is applied, accounting for the known sensitivity and specificity of the screening tool [29,30].

In this study, AIMS-OD has a sensitivity of 94% and a specificity of 42%. True prevalence is the actual proportion of diseased persons in the study sample [31]. In our case, it is the true prevalence of positive patients taking into account the sensitivity and specificity of AIMS-OD. To estimate true prevalence of OD, the sensitivity (94%) and the specificity (42%) of AIMS-OD were taken into account by applying the following formula:


True prevalence=Estimated AIMS−OD prevalence+[(1−sensitivity)×(Estimated AIMS−OD prevalence)]−[(1−specificity)×(Estimated AIMS−OD prevalence)]

In this way, the true prevalence of OD patients is obtained, that is, without false positive diagnoses and with missing true positive diagnoses [30]. In the descriptive analyses, dichotomous data were presented in the results tables as relative and absolute frequencies, corresponding to analysis with chi-square test [32], due to the large size of the samples. Continuous data were presented in tables as mean and SD. The mortality variables do not include patients who died in the previous time period, that is, the patients who died in the monthly variable are those who died in the 30 days following discharge. The total mortality variable does not correspond to the sum of the mortality variables, since there are patients with a death date after 1 year. The comparison analysis between samples was performed using the 2-tailed *t* test (between-group comparisons) [33] and the nonparametric Mann-Whitney *U* test [34] was used for those variables that did not follow a normal distribution (comparison between 2 groups). To perform the 3-sample comparison analysis, the Kruskal-Wallis test [35] was used.

A comparative analysis was also carried out between the variables of 2 groups, R13-OD patients versus H-AIMS-OD patients and the union of the 2 previous groups versus patients with no R13-OD and L-AIMS-OD. For those variables that did not follow a normal distribution, a comparative analysis was performed with the nonparametric Mann-Whitney *U* test (between-group comparisons). For the analysis of normality of variables, we used Shapiro’s normality test to evaluate the normality of a single variable within a group, and Levene’s test to evaluate the equality of variances between the 3 groups studied.

In the bivariate analysis, Fisher exact test was used to assess the relationships between the different OD factors and elevated risk of AIMS-OD. For continuous factors, Student *t* test (normal distribution) and Mann-Whitney *U* test (nonnormal distribution) were used. Multivariate models were performed with the significantly associated factors (*P*<.05) and clinically relevant factors for the different outcomes by numerical normalization of the data and logistic regression. Comparative plots of prevalences of R13-OD and H-AIMS-OD were created for each age range. The results were interpreted according to the *P* value obtained, the probability of occurrence of the outcome (odds ratio), the magnitude of the observed effect, and its clinical and biological plausibility. Statistical significance was accepted if *P* values were <.05. Statistical analysis was performed with the specific language R and Python.

## Results

### Demographics and Clinical Characteristics of the Overall Study Population

A total of 257,541 patients with COVID-19 disease were studied, divided into 152,643 patients in the group aged 18‐69 years, and 104,898 patients in the group aged 70 years and older. Prevalence of AP, MN, and DH was higher in patients aged 70 years and older. Weight was lower but BMI was higher in patients aged 70 years and older than in the whole population or patients aged 18‐69 years. The main clinical characteristics of the sample by age groups are shown in Table 2.

**Table 2.** Clinical and descriptive conditions of the total samples for patients aged 18 years and older and for the 18‐69 years and 70 years and older subgroups. *P* values in italics indicate statistical differences, and the unitalicized *P* values nearly reach statistical significance.

Characteristics	Total (18 years and older; N=257,541)	18‐69 years (N=152,643)	70 years and older (N=104,898)	*P* value
Age (years), mean (SD)	62.0 (20.1)	48.3 (13.6)^a^^,b^	81.9 (7.4)^a^	*<.001*
Sex (female), n (%)	146,474 (56.9)	88,525 (58.0)^a^^,b^	57,949 (55.2)^a^	*<.001*
Length of stay (days), mean (SD)	10.5 (20.1)	10.3 (16.7)^a^^,b^	10.7 (22.4)^a^	*.04*
Charlson, mean (SD)	1.5 (2.4)	0.9 (2.0)^a^	2.2 (2.7)^a^	*<.001*
Aspiration pneumonia, n (%)	3666 (1.4)	827 (0.5)^a^^,b^	2839 (2.7)^a^	*<.001*
Malnutrition, n (%)	3140 (1.2)	1165 (0.8)^a^^,b^	1975 (1.9)^a^	*<.001*
Dehydration, n (%)	3391 (1.3)	715 (0.5)^a^^,b^	2676 (2.6)^a^	*<.001*
Discharge to nursing home, n (%)	20,362 (7.9)	6060 (4.0)^a^^,b^	14,302 (13.6)^a^	*<.001*
ICU^c^ admissions, n (%)	10,431 (4.1)	6301 (4.1)^d^	4130 (3.9)	.054
ICU days, mean (SD)	7.6 (15.2)	8.7 (13.4)^a^^,b^	6.0 (10.1)^a^	*<.001*
Weight (kg), mean (SD)	76.8 (22.3)	76.9 (22.8)	75.4 (16.5)	*.02*
BMI, mean (SD)	28.4 (10.7)	28.2 (10.5)^e^	30.1 (11.5)^f^	.05
Total mortality, n (%)	9926 (3.9)	1204 (0.8)^a^^,b^	8722 (8.3)	*<.001*
Mortality during hospital stay, n (%)	4892 (1.9)	840 (0.6)^a^^,b^	4052 (3.9)^a^	*<.001*
One-month mortality, n (%)	4610 (1.8)	494 (0.3)^a^^,b^	4116 (3.9)^a^	*<.001*
Six-month mortality, n (%)	1238 (0.5)	134 (0.1)^a^^,b^	1104 (1.1)^a^	*<.001*
One-year mortality, n (%)	87 (0.03)	10 (0.007)^a^^,b^	77 (0.07)^a^	*<.001*

a *P*<.001 vs Total ≥18 years.

b *P*<.001 vs ≥70 years.

c ICU: intensive care unit.

d *P*<.05 vs ≥70 years.

e *P*<.01 vs ≥70 years.

f *P*<.05 vs Total ≥18 years.

### Prevalence of OD in the Overall Study Population and in Hospitalization Versus Primary Care Population According to *ICD* Codes Versus AIMS-OD

The overall prevalence of R13-OD for those aged 18 years and older was 4.6%; for those aged 18‐69 years, 2.4%; and for those aged 70 years and older, 7.7%. In contrast, AIMS-OD estimated a prevalence of 25.5% for those aged 18 years and older, of 20.8% for those aged 18‐69 years, and of 32.3% for those aged 70 years and older. Calculated true prevalences for the study groups were 12.2%, 10.0%, and 15.5%, respectively.

**Figure 2.**
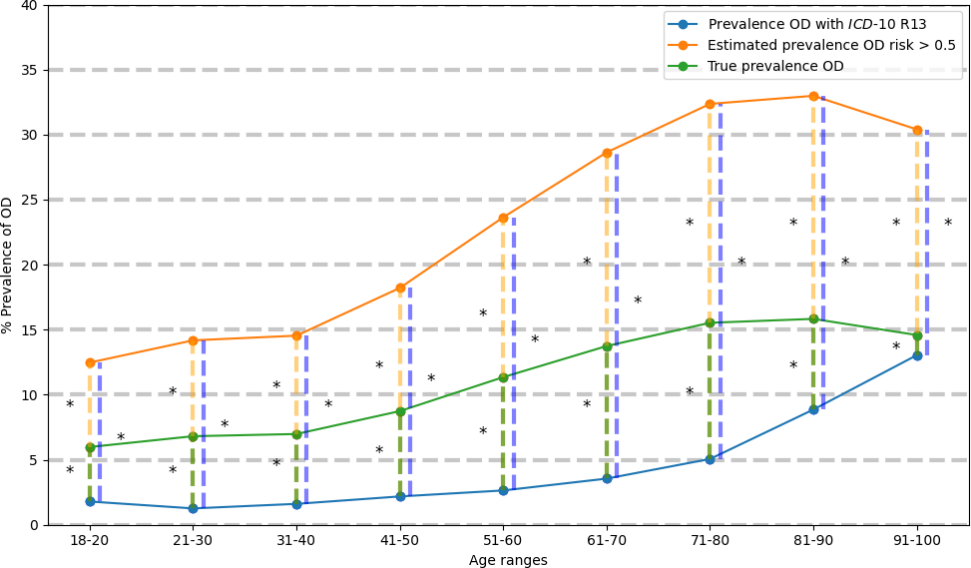
Prevalence of total patients aged 18 years and older with oropharyngeal dysphagia (OD) by 10-year age ranges. The orange line shows the estimated prevalence of H-AIMS-OD, the green line shows the true prevalence of OD, and the blue line shows the prevalence of R13-OD for patients aged 18 years and older. The orange dashed line shows the comparison between the estimated prevalence of high risk of OD versus the true prevalence of OD in each age range. The green dashed line shows the comparison between the true prevalence of OD versus the R13-OD prevalence in each age range. The blue dashed line shows the comparison between the estimated prevalence of H-AIMS-OD versus R13-OD prevalence in each age range. These comparisons are represented with *P<.001. *ICD-10*: *International Statistical Classification of Diseases, Tenth Revision*; OD: oropharyngeal dysphagia.

As shown in Figure 2, in the group of patients in R13-OD, prevalence of OD increased significantly from 11 to 100 years to reach an OD prevalence of 13.0%. In those at H-AIMS-OD, OD increased between the age range of 71‐80 and 81‐90 years to reach a prevalence of 33.0% and then decreased. In contrast, when true prevalence was taken into account, OD increased between 61‐70 and 81‐90 years to reach a prevalence of 15.8% and then decreased.

The prevalence in each age range in patients in R13-OD versus patients in H-AIMS-OD was statistically significantly different. The same is true between the groups of patients in R13-OD versus the True prevalence according to AIMS-OD psychometrics, and between the groups of patients in H-AIMS-OD versus the True prevalence (Figure 2). The mean prevalence of hospitalized patients in R13-OD was 3.5%; in H-AIMS-OD, 34.8%; and according to the calculated true prevalence, 16.7%.

**Figure 3.**
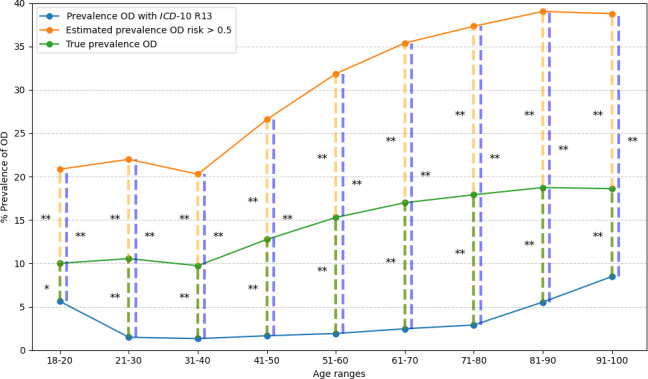
Prevalence of hospitalized patients aged 18 years and older with oropharyngeal dysphagia (OD) by 10-year age ranges. The orange line shows the estimated prevalence of high risk of OD (AIMS-OD > 0.5), the green line shows the true prevalence of OD, and the blue line shows the prevalence of R13-OD for hospitalized patients aged 18 years and older. The orange dashed line shows the comparison between the estimated prevalence of H-AIMS-OD versus the true prevalence of OD in each age range. The green dashed line shows the comparison between the true prevalence of OD versus R13-OD prevalence in each age range. The blue dashed line shows the comparison between the estimated prevalence of H-AIMS-OD versus the R13-OD prevalence in each age range. These comparisons are represented with *P<.05, ** *P*<.001. *ICD-10*: *International Statistical Classification of Diseases, Tenth Revision*; OD: oropharyngeal dysphagia.

The prevalence in each age range in hospitalized patients in each of the 3 groups was significantly different (Figure 3).

The mean prevalence of primary care in patients in the R13-OD group was 4.3%; in H-AIMS-OD, 15.4%; and according to the calculated true prevalence, 7.4%.

**Figure 4.**
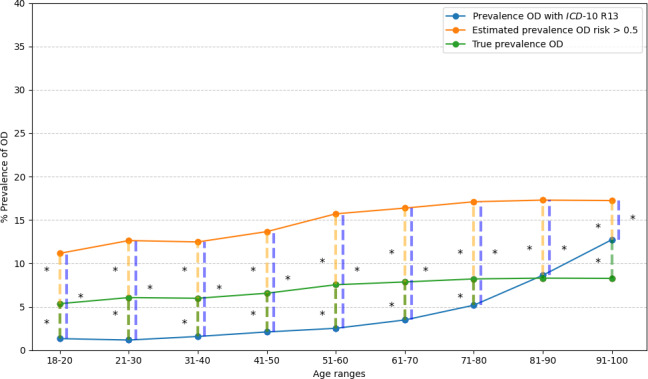
Prevalence of primary care patients aged 18 years and older with oropharyngeal dysphagia (OD) by 10-year age ranges. The orange line shows the estimated prevalence of high risk of OD (AIMS-OD>0.5), the green line shows the true prevalence of OD, and the blue line shows the prevalence of R13-OD for hospitalized patients aged 18 years and older. The orange dashed line shows the comparison between the estimated prevalence of H-AIMS-OD versus the true prevalence of OD in each age range. The green dashed line shows the comparison between the true prevalence of OD versus R13-OD prevalence in each age range. The blue dashed line shows the comparison between the estimated prevalence of H-AIMS-OD versus R13-OD prevalence in each age range. These comparisons are represented with *P<.001. *ICD-10*: *International Statistical Classification of Diseases, Tenth Revision*; OD: oropharyngeal dysphagia.

The prevalence in each age range in primary care patients in each of the 3 groups was significantly different except in the 81‐90 years age group between the prevalence of patients in R13-OD versus patients in H-AIMS-OD (Figure 4).

### Clinical Characteristics and Outcomes of the Patients in R13-OD Versus AIMS-OD Status

#### Overall Study Population

Patients in R13-OD in all 3 samples had higher rates of AP, MN, DH, hospital stay, and total mortality than those in H-AIMS-OD, and these, in turn, were higher than those with no R13-OD and L-AIMS-OD. In contrast, in patients aged 70 years and older, institutionalization was higher in H-AIMS-OD patients, and Charlson was also higher in H-AIMS-OD patients of the 3 patient groups. Of all groups, weight was lower in R13-OD patients. The main clinical characteristics according to age groups and R13-OD or AIMS-OD subgroups are shown in Table 3.

**Table 3.** Clinical conditions for patients aged 18 years and older, those between 18 and 69 years, and those aged 70 years and older according to OD^a^ classification (R13-OD; H-AIMS-OD, and no R13-OD and L-AIMS-OD)^b^.

Characteristics	Total (18 years and older)				18‐69 years				70 years and older			
R13-OD	H-AIMS-OD	No R13-OD and L-AIMS-OD	*P* value	R13-OD	H-AIMS-OD	No R13-OD and L-AIMS-OD	*P* value	R13-OD	H-AIMS-OD	No R13-OD and L-AIMS-OD	*P* value
Participants, n/N (%)	11,744/257,541 (4.6)	65,576/257,541 (25.5)	183,739/257,541 (71.3)		3649/152,643 (2.4)	31,698/152,643 (20.8)	118,181/152,643 (77.4)		8095/104,898 (7.7)	33,878/104,898 (32.3)	65,558/104,898 (62.5)	
Age, mean (SD)	74.7 (17.7)	67.3 (18.3)	59.6 (20.3)	<.001^c^; <.001^c^	52.4 (12.7)	51.8 (12.9)	47.3 (13.6)	.005^c^; <.001^c^	84.8 (7.4)	81.9 (7.3)	82.0 (7.5)	<.001^c^; <.001^c^
Sex (female), n (%)	6571 (56.0)	34,104 (52.0)	107,596 (58.6)	<.001^c^; <.001^c^	1879 (51.5)	16,522 (52.1)	70,505 (59.7)	.50; <.001^c^	4692 (58.0)	17,582 (51.9)	37,091 (56.6)	<.001^c^; <.001^c^
Length of stay, mean (SD)	15.5 (19.7)	10.9 (25.7)	10.0 (15.0)	<.001^c^; <.001^c^	19.9 (27.3)	10.8 (17.7)	9.8 (15.7)	<.001^c^; <.001^c^	14.1 (16.5)	10.9 (29.7)	10.2 (14.4)	<.001^c^; <.001^c^
Charlson, mean (SD)	1.7 (2.7)	2.1 (2.8)	1.2 (2.2)	<.001^c^; <.001^c^	0.9 (2.0)	1.4 (2.5)	0.8 (1.8)	<.001^c^; <.001^c^	2.2 (2.7)	2.7 (2.9)	2.0 (2.5)	<.001^c^; <.001^c^
Aspiration pneumonia, n (%)	1255 (10.7)	1506 (2.3)	1455 (0.8)	<.001^c^; <.001^c^	213 (5.8)	307 (1.0)	395 (0.3)	<.001^c^; <.001^c^	1042 (12.9)	1199 (3.5)	1060 (1.6)	<.001^c^; <.001^c^
Malnutrition, n (%)	491 (4.2)	1312 (2.0)	1564 (0.9)	<.001^c^; <.001^c^	133 (3.6)	434 (1.4)	659 (0.6)	<.001^c^; <.001^c^	358 (4.4)	878 (2.3)	905 (1.4)	<.001^c^; <.001^c^
Dehydration, n (%)	441 (3.8)	1391 (2.1)	1751 (1.0)	<.001^c^; <.001^c^	50 (1.4)	268 (0.8)	422 (0.4)	.001^c^; <.001^c^	391 (4.8)	1123 (3.3)	1329 (2.0)	<.001^c^; <.001^c^
Discharge to nursing home, n (%)	1762 (15.0)	8501 (13.0)	(10,867 (5.9)	<.001^c^; <.001^c^	329 (9.0)	2251 (7.1)	3610 (3.1)	<.001^c^; <.001^c^	1433 (17.7)	6250 (18.4)	7257 (11.1)	.12; <.001^c^
ICU^d^ admissions, n (%)	433 (3.7)	4095 (6.2)	6101 (3.3)	<.001^c^; <.001^c^	234 (6.4)	2328 (7.3)	3839 (3.3)	.04^c^; <.001^c^	199 (2.5)	1767 (5.2)	2262 (3.5)	<.001^c^; <.001^c^
ICU days, mean (SD)	12.6 (17.5)	7.8 (12.4)	7.3 (11.8)	<.001^c^; .88	15.6 (18.9)	8.9 (13.5)	8.3 (13.0)	<.001^c^; .13	9.2 (15.1)	6.3 (10.7)	5.7 (9.3)	.13; .04^c^
Weight, mean (SD)	73.2 (15.9)	77.2 (17.6)	76.8 (22.7)	<.001^c^; *.05*	74.7 (16.1)	78.7 (19.0)	76.9 (23.0)	.003^c^; .03^c^	68.7 (14.4)	76.0 (16.4)	75.7 (16.6)	<.001^c^; .90^c^
BMI, mean (SD)	28.1 (14.4)	28.9 (9.1)	28.3 (10.8)	*.06; .07*	27.3 (14.0)	28.1 (6.6)	28.2 (10.6)	.87; .82	29.0 (15.1)	31.1 (10.6)	29.7 (10.8)	.009^c^; .89
Total mortality, n (%)	1207 (8.7)	3617 (5.5)	5646 (3.1)	<.001^c^; <.001^c^	71 (1.9)	430 (1.4)	731 (0.6)	.004^c^; <.001^c^	956 (11.8)	3187 (9.4)	4915 (7.5)	<.001^c^; <.001^c^
Mortality during hospital stay, n (%)	350 (3.0)	2139 (3.3)	2568 (1.4)	.11; <.001^c^	45 (1.2)	318 (1.0)	497 (0.4)	.19; <.001^c^	305 (3.8)	1821 (5.4)	2071 (3.2)	<.001^c^; <.001^c^
One-month mortality, n (%)	333 (2.8)	863 (1.3)	1560 (0.8)	<.001^c^; <.001^c^	13 (0.4)	65 (0.2)	125 (0.1)	.01^c^; <.001^c^	320 (4.0)	798 (2.4)	1435 (2.2)	<.001^c^; <.001^c^
Six-month mortality, n (%)	122 (1.0)	413 (0.6)	527 (0.3)	<.001^c^; <.001^c^	16 (0.2)	43 (0.1)	73 (0.06)	.36; <.001^c^	116 (1.4)	370 (1.1)	454 (0.7)	<.001^c^; <.001^c^
One-year mortality, n (%)	9 (0.08)	30 (0.05)	42 (0.02)	.14; <.001^c^	0 (0.0)	2 (0.006)	5 (0.004)	.60; .01^c^	9 (0.1)	28 (0.08)	37 (0.06)	.46; *.05*

a OD: oropharyngeal dysphagia.

b In each *P* value row, the first *P *value is the comparison between the group of R13-OD patients and the group of H-AIMS-OD patients. The second *P* value is the comparison between the union of the first groups (R13-OD and H-AIMS-OD) versus the group of patients with no R13-OD and L-AIMS-OD.

c These values indicate statistical differences (*P* values), and values in italics nearly reach statistical significance.

d ICU: intensive care unit.

#### Hospitalized Patients

A total of 108,039 patients were admitted to hospital, of whom 44.3% (n=47,841) were aged between 18 and 69 years and 55.7% (n=60,198) were aged 70 years and older.

Hospitalized patients aged 70 years and older had a mean age of 81.5 (SD 7.1) years (*P*<.001); had a total mortality of 10.5% (6320/60,198; *P*<.001); prevalence of AP of 4.0% (2407/60,198; *P*<.001), MN of 3.1% (1866/60,198; *P*<.001), and DH of 3.8% (2287/60,198; *P*<.001); and mean weight of 76.9 (SD 16.7) kg (*P*=.002).

As shown in Table 4, inpatient populations aged 18 years and older, 18‐69 years, and 70 years and older were analyzed, according to R13-OD, H-AIMS-OD, no R13-OD, and L-AIMS-OD. We found that the prevalence of OD in patients aged 18 years and older was 3.5% (3753/108,039) in R13-OD and 34.8% (37,618/108,039) in H-AIMS-OD with a true prevalence of 16.7% (18,042/108,039), with 63.0% (68,053/108,039) of patients in this age group with no R13-OD and L-AIMS-OD. Patients aged 18‐69 years had a prevalence of 2.0% (965/47,841) in R13-OD, 30.6% (14,645/47,841) in H-AIMS-OD, with a True prevalence of 14.7% (7032/47,841), with 68.0% (62,552/47,841) of patients in this age group with no R13-OD and L-AIMS-OD. Patients aged 70 years and older had a prevalence of OD of 4.6% (2788/60,198; R13-OD), and of 38.2% (22,973/60,198; H-AIMS-OD) with a True prevalence of 18.3% (11,016/60,198), with 59.0% (35,501/60,198) of patients in this age group with no R13-OD and L-AIMS-OD.

The main clinical characteristics of the hospitalized groups are shown in Table 4.

**Table 4.** Clinical conditions of the hospitalization samples and comparison between R13-OD individuals, H-AIMS-OD, and those with no R13-OD and L-AIMS-OD^a^.

Characteristics	Hospitalized patients aged 18 years and older				18‐69 years				70 years and older			
R13-OD^b^	H-AIMS-OD	No R13-OD and L-AIMS-OD	*P* value	R13-OD	H-AIMS-OD	No R13-OD and L-AIMS-OD	*P* value	R13-OD	H-AIMS-OD	No R13-OD and L-AIMS-OD	*P* value
Participants, n/N (%)	3753/108,039 (3.5)	37,618/108,039 (34.8)	68,053/108,039 (63.0)		965/47,841 (2.0)	14,645/47,841 (30.6)	32,552/47,841 (68.0)		2788/60,198 (4.6)	22,973/60,198 (38.2)	35,501/60,198 (59.0)	
Age, mean (SD)	77.1 (15.2)	71.7 (15.2)	67.9 (17.0)	*<.001; <.001*	55.7 (11.8)	56.1 (10.7)	53.3 (11.9)	.70; *<.001*	84.5 (7.1)	81.7 (7.1)	81.3 (7.1)	*<.001; <.001*
Sex (female), n (%)	1694 (45.1)	17,200 (45.7)	31,434 (46.2)	.49; .09	307 (31.8)	6051 (41.3)	14,076 (43.2)	*<.001*; *<.001*	1387 (49.7)	11,149 (48.5)	17,358 (48.9)	.22; .39
Length of stay, mean (SD)	14.2 (17.2)	9.0 (24.9)	8.4 (12.7)	*<.001; <.001*	18.5 (24.4)	8.9 (13.3)	8.4 (13.8)	*<.001; <.001*	12.8 (13.5)	9.0 (30.0)	8.4 (11.5)	*<.001; <.001*
Charlson, mean (SD)	3.2 (3.3)	3.0 (3.0)	3.0 (3.0)	*<.001; <.001*	2.0 (3.1)	2.1 (3.0)	1.9 (2.9)	*.009; <.001*	3.6 (3.3)	3.2 (3.0)	3.2 (3.0)	*<.001;* *.003*
Aspiration pneumonia, n (%)	907 (24.2)	1149 (3.1)	1398 (2.1)	*<.001; <.001*	162 (16.8)	241 (1.6)	377 (1.2)	*<.001; <.001*	745 (26.7)	908 (4.0)	1021 (2.9)	*<.001; <.001*
Malnutrition, n (%)	399 (10.6)	1116 (3.0)	1682 (2.5)	*<.001; <.001*	108 (11.2)	380 (3.0)	706 (2.2)	*<.001; <.001*	291 (10.4)	736 (3.2)	976 (2.7)	*<.001*; .*001*
Dehydration, n (%)	301 (8.0)	1115 (3.0)	1667 (2.4)	*<.001; <.001*	40 (4.1)	222 (1.5)	415 (1.3)	*<.001;* *<.001*	261 (9.4)	893 (3.9)	1252 (3.5)	*<.001; .023*
Discharge to nursing home, n (%)	1297 (34.6)	7371 (19.6)	12,153 (17.9)	*<.001; <.001*	282 (29.2)	1947 (13.3)	3927 (12.1)	*<.001; <.001*	1015 (36.4)	5424 (23.6)	8226 (23.2)	*<.001*; .22
ICU^c^ admissions, n (%)	330 (8.8)	3536 (9.4)	6692 (9.8)	.22; *.008*	185 (19.2)	2002 (13.7)	4176 (12.8)	<*.001; <.001*	145 (5.2)	1534 (6.7)	2516 (7.1)	.22; .06
ICU days, mean (SD)	14.6 (18.7)	7.9 (12.4)	7.2 (11.8)	*<.001; .01*	17.7 (19.9)	9.0 (13.3)	8.2 (13.1)	*<.001; .02*	10.7 (16.3)	6.5 (11.0)	5.6 (9.1)	*.01; .04*
Weight, mean (SD)	77.1 (16.6)	77.3 (16.7)	80.7 (18.9)	.70; *<.001*	79.8 (17.4)	83.4 (17.3)	81.0 (19.1)	.14; .53	72.3 (14.2)	76.4 (16.5)	77.5 (16.9)	.17; .26
BMI, mean (SD)	26.1 (8.9)	31.8 (10.6)	30.2 (11.8)	.10; .34	24.5 (13.3)	31.8 (4.7)	30.2 (11.7)	.29; .44	27.4 (2.8)	31.8 (11.4)	30.4 (12.4)	.14; .48
Total mortality, n (%)	416 (11.1)	2847 (7.6)	4255 (6.3)	*<.001; <.001*	52 (5.4)	369 (2.5)	661 (2.0)	*<.001; <.001*	364 (13.1)	2478 (10.8)	3594 (10.1)	*<.001; <.001*
Mortality during admission, n (%)	229 (6.1)	1906 (5.1)	2832 (4.2)	*<.001;* .31	41 (4.2)	289 (2.0)	523 (1.6)	.93; .77	188 (6.7)	1617 (7.0)	2309 (6.5)	*<.001;* .54
One-month mortality, n (%)	102 (2.7)	555 (1.5)	851 (1.3)	*.02;* .88	7 (0.7)	50 (0.3)	84 (0.3)	.98; .69	95 (3.4)	505 (2.2)	767 (2.2)	*.01;* .82
Six-month mortality, n (%)	80 (2.1)	361 (1.0)	532 (0.8)	*<.001;* .19	4 (0.4)	28 (0.2)	47 (0.1)	.98; .76	76 (2.7)	333 (1.4)	485 (1.4)	<*.001;* .30
One-year mortality, n (%)	4 (0.1)	21 (0.1)	32 (0.05)	.62; .94	0 (0.0)	2 (0.01)	4 (0.01)	.59; .78	4 (0.14)	19 (0.08)	28 (0.08)	.50; .89

a In each *P* value row, the first *P* value is the group of R13-OD patients compared with the group of H-AIMS-OD patients. The second *P* value is the comparison between the union of the first groups (R13-OD and H-AIMS-OD) versus the group of patients with no R13-OD and L-AIMS-OD. Values in italics indicate statistical differences (*P* values).

b OD: oropharyngeal dysphagia.

c ICU: intensive care unit.

#### Primary Care Patients

A total of 206,151 patients were visited in primary care, of whom 62.4% (n=128,714) were aged between 18 and 69 years and 37.6% (n=77,437) were aged 70 years and older. Primary care patients aged 70 years and older had a mean age of 82.2 (SD 7.5) years (*P*<.001), a prevalence of total mortality of 7.3% (5,652/77,437; *P*<.001), a prevalence of AP of 0.8% (619/77,437; *P*<.001), MN of 0.1% (77/77,437; *P*<.001), and DH of 0.6% (464/77,437; *P*<.001), and mean weight was 74.8 (SD 16.5) kg (*P*=.002).

The 3 age groups in the primary care population were analyzed according to OD diagnosis. In patients aged 18 years and older, we found that the prevalence of OD in the R13-OD group was 4.3% (8863/206,151); in H-AIMS-OD patients, 15.4% (31,749/206,151); and True prevalence, 7.39% (15,234/206,151), with 81.0% (167,033/206,151) of patients from this age group with no R13-OD and L-AIMS-OD. Patients aged between 18 and 69 years had a prevalence of OD in the R13-OD group of 2.2% (2879/128,714); in the H-AIMS-OD group, 14.3% (18,411/128,714) and True prevalence, 6.9% (8881/128,714), with 83.8% (107,864/128,714) of patients from this age group with no R13-OD and L-AIMS-OD. Patients aged 70 years and older from the R13-OD group had a prevalence of OD of 7.7% (5984/77,437), H-AIMS-OD patients had an estimated prevalence of 17.2% (13,338/77,437), and True prevalence was 8.3% (6427/77,437), with 76.4% (59,169/77,437) of patients from this age group with no R13-OD and L-AIMS-OD. In the hospitalized population, the prevalence of OD, AP, MN, and DH was higher than in primary care, except for OD for those aged 70 years and older. The main clinical characteristics of the primary care groups are shown in Table 5.

**Table 5.** Clinical conditions of the primary care groups and comparison between R13-OD, H-AIMS-OD, and those with no R13-OD and L-AIMS-OD^a^.

Characteristics	Primary care patients aged 18 years and older				18‐69 years				70 years and older			
	R13-OD^b^	H-AIMS-OD	NO R13-OD and L-AIMS-OD	*P* value	R13-OD	H-AIMS-OD	NO R13-OD and L-AIMS-OD	*P* value	R13-OD	H-AIMS-OD	NO R13-OD and L-AIMS-OD	*P* value
Participants, n/N (%)	8863/206,151 (4.3)	31,749/206,151 (15.4)	167,033/206,151 (81.0)		2879/128,714 (2.2)	18,411/128,714 (14.3)	107,864/128,714 (83.8)		5984/77,437 (7.7)	13,338/77,437 (17.2)	59,169/77,437 (76.4)	
Age (years), mean (SD)	74.1 (18.3)	62.8 (20.0)	59.3 (20.5)	*<.001; <.001*	51.7 (12.7)	48.7 (13.4)	46.9 (13.7)	*<.001; <.001*	84.9 (7.5)	82.2 (7.5)	81.9 (7.4)	*<.001;* .56
Sex (female), n (%)	5270 (59.5)	18,644 (58.7)	99,914 (59.8)	.21; *<.001*	1629 (56.6)	11,040 (60.0)	65,937 (61.1)	*<.001; <.001*	3641 (60.8)	7604 (57.0)	33,977 (57.4)	*<.001;* .12
Charlson, mean (SD)	0.4 (1.0)	0.6 (1.1)	0.6 (1.1)	*<.001; .007*	0.3 (0.8)	0.5 (1.0)	0.5 (1.0)	*<.001;* .07	0.5 (1.0)	0.9 (1.2)	0.9 (1.2)	*<.001; <.001*
Aspiration pneumonia, n (%)	200 (2.3)	138 (0.4)	445 (0.3)	*<.001; <.001*	31 (1.1)	33 (0.2)	94 (0.09)	*<.001; <.001*	169 (2.8)	105 (0.8)	351 (0.6)	<*.001;* .88
Malnutrition, n (%)	25 (0.3)	14 (0.04)	69 (0.04)	*<.001; <.001*	6 (0.2)	1 (0.005)	8 (0.007)	*<.001; .002*	19 (0.3)	13 (0.1)	61 (0.1)	*<.001;* .54
Dehydration, n (%)	43 (0.5)	83 (0.3)	379 (0.2)	*<.001; .002*	2 (0.07)	7 (0.04)	57 (0.05)	.46; .53	41 (0.7)	76 (0.6)	322 (0.5)	.34; .89
Weight, mean (SD)	72.3 (15.5)	77.5 (18.4)	76.4 (23.0)	*<.001;* .49	73.8 (15.6)	77.8 (19.1)	76.6 (23.4)	*.01;* .70	67.3 (14.2)	76.8 (16.7)	75.2 (16.5)	*<.001;* .06
BMI, mean (SD)	28.7 (15.2)	27.6 (6.0)	28.1 (10.2)	.69; .56	28.0 (14.4)	27.4 (6.7)	28.0 (10.4)	.67; .89	29.4 (16.4)	28.3 (3.9)	28.9 (8.2)	.23; .72
Total mortality, n (%)	701 (7.9)	1037 (3.3)	4627 (2.8)	*<.001; <.001*	30 (1.0)	79 (0.4)	498 (0.5)	*<.001;* .33	671 (11.2)	958 (7.2)	4129 (7.0)	*<.001;* .65
One-month mortality, n (%)	350 (3.9)	607 (1.9)	2818 (1.7)	*<.001; <.001*	17 (0.6)	52 (0.3)	334 (0.3)	.38; .45	333 (5.6)	555 (4.2)	2484 (4.2)	*<.001; <.001*
Six-month mortality, n (%)	37 (0.4)	67 (0.2)	295 (0.2)	.30; .57	1 (0.03)	5 (0.03)	61 (0.06)	.55; *.04*	36 (0.6)	62 (0.5)	234 (0.4)	.36; *.61*
One-year mortality, n (%)	6 (0.07)	12 (0.04)	35 (0.02)	.54; .27	0 (0.0)	1 (0.005)	5 (0.005)	.55; .93	6 (0.1)	11 (0.08)	30 (0.05)	.62; *.22*

a In each *P* value row, the first *P* value is the group of R13-OD patients compared with the group of H-AIMS-OD patients. The second *P* value is the comparison between the union of the first groups (R13-OD and H-AIMS-OD) versus the group of patients with no R13-OD and L-AIMS-OD. Values in italics indicate statistical differences (*P* values).

b OD: oropharyngeal dysphagia.

### Risk Factors for OD From Bivariate and Multivariate Analysis

#### Risk Factors for All Patients

For the bivariate analysis, the risk factors in the total population for the *ICD-10* R13 variable and risk >0.5 according to AIMS-OD, there is one that differs: in the results of the dependent variable risk of >0.5, the main risk factors were respiratory diseases such as chronic obstructive pulmonary disease. On the other hand, in the results of the multivariate analysis of the dependent variable *ICD-10* R13, the main risk factors were age, the use of neuroleptics, and cerebral infarction, the same as for the dependent variable risk >0.5 according to AIMS-OD (Tables6  7).

**Table 6.** The main risk factors studied in the bivariate and multivariate analysis of the total hospitalized and primary care populations according to the dependent variable *ICD-10* R13, with the *P* values and odds ratio^a^.

Total patients						Hospitalized patients						Primary care patients					
Bivariate analysis			Multivariate analysis			Bivariate analysis			Multivariate analysis			Bivariate analysis			Multivariate analysis		
	*P* value	OR^b^	Variable	*P* value	OR	Variable	*P* value	OR	Variable	*P* value	OR	Variable	*P* value	OR	Variable	*P* value	OR
Age >70 years	*<.0001*	3.31	Age >70 years	*<.0001*	1.74	Age >70 years	*<.0001*	2.37	Age >70 years	*<.0001*	1.62	Age >70 years	*<.0001*	3.67	N/A^c^	N/A	N/A
Trazodone	*<.0001*	3.20	Trazodone	*.03*	1.20	Trazodone	*<.0001*	2.80	Trazodone	*<.0001*	1.53	N/A	N/A	N/A	N/A	N/A	N/A
Cerebral infarction	*<.0001*	2.55	Cerebral infarction	*<.0001*	1.96	Delirium	*<.0001*	3.75	Delirium	*<.0001*	1.55	N/A	N/A	N/A	N/A	N/A	N/A
Institutionalization	*<.0001*	2.20	Institutionalization	*<.0001*	1.63	Institutionalization	*<.0001*	2.41	Institutionalization	*<.0001*	1.49	N/A	N/A	N/A	N/A	N/A	N/A
Citalopram	*.0001*	2.04	Vitamin D deficiency	*<.0001*	1.41	Dementia	*<.0001*	4.00	Dementia	*<.0001*	1.69	N/A	N/A	N/A	N/A	N/A	N/A
Acute renal failure	*<.0001*	1.95	Acute renal failure	*<.0001*	1.26	Acute renal failure	*<.0001*	2.03	Acute renal failure	*<.0001*	1.32	N/A	N/A	N/A	N/A	N/A	N/A
Sertraline	*<.0001*	1.90	Deficiency of other B vitamins	*<.0001*	1.27	Admissions	*<.0001*	1.14	Type 2 diabetes mellitus with hyperglycemia	*.04*	1.28	N/A	N/A	N/A	N/A	N/A	N/A
Hypertensive renal disease	*<.0001*	1.77	Pneumonia caused by microorganisms	*<.0001*	1.30	Alzheimer disease	*<.0001*	3.76	Pneumonia caused by microorganisms	*<.0001*	1.37	N/A	N/A	N/A	N/A	N/A	N/A
Depressive episode	*<.0001*	1.44	Mirtazapine	*<.0001*	2.29	Mirtazapine	*<.0001*	2.05	Mirtazapine	*<.0001*	1.33	N/A	N/A	N/A	N/A	N/A	N/A
Chronic kidney disease	*<.0001*	1.39	Acute bronchitis	*<.0001*	1.16	Stays	*<.0001*	1.01	N/A	N/A	N/A	N/A	N/A	N/A	N/A	N/A	N/A
COPD^d^	*<.0001*	1.27	COPD	*.04*	1.10	Charlson	*<.0001*	1.05	N/A	N/A	N/A	N/A	N/A	N/A	N/A	N/A	N/A

a Values in italics indicate a significant association between the dependent variable and the factor.

b OR: odds ratio.

c N/A: not applicable.

d COPD: chronic obstructive pulmonary disease.

**Table 7.** The main risk factors studied in the bivariate and multivariate analysis of the total hospitalization and primary care populations according to the dependent variable risk >0.5, with the *P* values and odds ratios^a^.

Total patients						Hospitalized patients						Primary care patients					
Bivariate analysis			Multivariate analysis			Bivariate analysis			Multivariate analysis			Bivariate analysis			Multivariate analysis		
Variable	*P* value	OR^b^	Variable	*P* value	OR	Variable	*P* value	OR	Variable	*P* value	OR	Variable	*P* value	OR	Variable	*P* value	OR
COPD^c^	*<.0001*	1.40	Age >70 years	*<.0001*	1.30	Age >70 years	*<.0001*	1.40	Age >70 years	*<.0001*	1.17	Age >70 years	*<.0001*	1.25	Age >70 years	*<.0001*	1.16
COPD with exacerbation	*<.0001*	1.19	Mirtazapine	*<.0001*	1.30	Alzheimer disease	*<.0001*	1.24	Chronic obstructive pulmonary disease	*.04*	1.09	Mirtazapine	*<.0001*	1.14	N/A^d^	N/A	N/A
COPD with acute infection of the airways	*<.0001*	1.17	Cerebral infarction	*<.0001*	1.21	Dementia	*<.0001*	1.19	N/A	N/A	N/A	Chronic kidney disease	*<.0001*	1.11	N/A	N/A	N/A
Acute lower respiratory tract infection	*<.0001*	1.17	Pneumonia caused by microorganisms	*<.0001*	1.15	Delirium	*<.0001*	1.17	N/A	N/A	N/A	N/A	N/A	N/A	N/A	N/A	N/A
Pneumonia caused by microorganisms	*<.0001*	1.15	Acute bronchitis	*<.0001*	1.14	Chronic kidney disease	*<.0001*	1.17	N/A	N/A	N/A	N/A	N/A	N/A	N/A	N/A	N/A
Other types of pneumonia	*<.0001*	1.15	N/A	N/A	N/A	N/A	N/A	N/A	N/A	N/A	N/A	N/A	N/A	N/A	N/A	N/A	N/A

a Values in italics indicate a significant association between the dependent variable and the factor.

b OR: odds ratio.

c COPD: Chronic obstructive pulmonary disease.

d N/A: not applicable.

#### Hospitalized Patients

For the hospitalized population, for the bivariate analysis, there were more protective factors for the dependent variable *ICD-10* R13 than for risk >0.5, such as heart disease, myocardial infarction, diabetes, and anxiety. The variables hospital stays and admissions were risk factors for the dependent variable *ICD-10* R13, whereas for the dependent variable risk >0.5, the variables hospital stays, admissions, and ICU admission were protective factors (Tables6  7). The most relevant differences in the protective factors were that in the factors of the dependent variable *ICD-10* R13, hospital stays, admissions, and Charlson are risk factors, while in the factors of the dependent variable risk >0.5 according to AIMS-OD, they were clearly protective factors, together with admission to the ICU.

#### Primary Care Patients

For the primary care population, we observed that the main risk factors were age, the use of neuroleptics, and chronic kidney disease (Tables6  7).

The main risk factors for the total, hospitalized, and primary care populations with the dependent variable *ICD-10* R13 are shown in Table 6. The main risk factors for the same populations but with the dependent variable risk >0.5 according to AIMS-OD are shown in Table 7.

## Discussion

### Principal Findings

This study assessed 257,541 patients with COVID-19 disease during the first wave of the 2020 pandemic across Catalonia, representing 82.2% (257,541/313,310) of Catalan citizens diagnosed with COVID-19 disease then according to PADRIS database [36]. The overall prevalence of OD in R13-OD was 4.6% (11,744/257,541), in contrast to 12.2% based on the True AIMS-OD prevalence, both showing significant age-related differences. In hospitalized patients, R13-OD prevalence of OD was 3.5%, and the True AIMS-OD prevalence was 16.7%, also with significant age-related differences. Among primary care patients, R13-OD prevalence of OD was 4.3%, and the True AIMS-OD prevalence was 7.4%, with age-related differences except in the 81‐90 years age group. Taken together, our study suggests that the overall rate of OD underdiagnosis during the first wave of the pandemic in Catalonia was as high as 62.3%. Discrepancies between R13-OD–coded prevalence and actual prevalence are 7.6% in the general patient population, 13.2% in hospitalized patients, and 3.1% in primary care patients. In hospitalized patients, where the discrepancy is most pronounced, this underdiagnosis suggests that a significant number of cases are not being identified by conventional R13-OD coding. This finding is of concern, given the higher risk profile of hospitalized patients. These gaps suggest that relying solely on R13-OD coding may result in substantial underestimation of OD prevalence, which could affect patient outcomes and care strategies.

Patients with R13-OD diagnosis from all age categories had higher rates of AP, MN, and DH; longer hospital stays; and higher total mortality than patients with H-AIMS-OD; in turn, in this group, these complications were also higher than those with no R13-OD and L-AIMS-OD. However, in patients aged 70 years and older, institutionalization was higher in H-AIMS-OD, and the Charlson score was also higher in these patients. Risk factors for R13-OD in the entire population included age, neuroleptic use, and cerebral infarction. Risk factors related to H-AIMS-OD were similar to those in R13-OD. For hospitalized patients, higher risks related to delirium and dementia were observed, and for primary care patients, the only related risk factor was age.

Our entire patient population had a mean age of 62.0 (SD 20.1) years, 56.9% (146,474/257,541) were female, and had a Charlson score of 1.5 (SD 2.4). The mean age of the hospitalized patients in this study was 69.4 (SD 16.5) years. One of our previously published studies with a cohort of 605 patients hospitalized with COVID-19 disease during the first 3 waves of the pandemic also in Catalonia shows a similar mean average age of 69.2 (SD 17.3) years and a percentage of female sex of 49.9% (301/605) [37]. In several of our studies in patients with COVID-19 disease, mean average Charlson scores of 5.3 (SD 1.7) and 3.7 (SD 2.6) were reported, higher than those reported in our study [5,38]. This is because in our cohort, there are patients treated in primary care who are generally less severe than hospitalized patients.

The prevalence of OD in R13-OD in the total and hospitalized population was lower than in several of our studies with patients hospitalized with COVID-19 disease that found a prevalence of OD of 47% (1108/2359), 51.7% (106/205), and 65.4% (166/254) during the first 3 waves of the pandemic [37-39]. The prevalence of H-AIMS-OD for the total population was 25.5% (65,576/257,541) and for those hospitalized, 34.8%, which were closer to the prevalence of OD reported in those studies. This further shows the high rate of underdiagnosis of OD in hospital and primary care settings during the COVID-19 pandemic. In our study, the rate of underdiagnosis of OD, according to the calculated true prevalence, was 41.9% in primary care and 79.0% in hospitalized patients. We also found that the prevalence of OD in R13-OD in primary care patients was higher than that in hospitalized patients. The cause of this could be that patients in primary care centers were older and included nursing home residents who had previously been diagnosed with OD, while hospitalized patients in the first wave of the pandemic were younger, and the diagnosis of OD was more complicated and frequently not performed due to lack of awareness of OD or health care saturation and risk of infection during the COVID-19 pandemic [5,37,38].

Patients with R13-OD had worse clinical outcomes and higher rates of complications than patients with H-AIMS-OD, and these patients had worse outcomes than patients with no R13-OD or L-AIMS-OD. The increased complications in the R13 group are probably due to higher severity of dysphagia and more obvious signs of swallowing impairment which alerted clinicians to their condition. Patients diagnosed with OD according to *ICD-10* (R13-OD) probably represent the most clinically severe cases, since the coding of OD in routine clinical practice is usually motivated by clear symptoms, such as aspiration, severe complications, or advanced disease. This represents a recurring selection bias in underdiagnosed conditions such as OD or AP [40]. The gradient observed in the results (R13-OD>H-AIMS-OD>L-AIMS-OD) should be interpreted as reflecting different levels of severity along the dysphagia continuum. *ICD-10* coding captures only a small, highly selected subgroup of severe cases, while AIMS-OD identifies a broader at-risk population, including patients who do not yet have obvious symptoms but who have a clinically significant risk profile. H-AIMS-OD patients experience clinical outcomes ranging between R13-OD and L-AIMS-OD patients, which is consistent with the role of screening tools: to detect risk earlier and more broadly than routine clinical diagnosis. A recent study using AIMS-OD to assess the prevalence of AP found similar patterns, in which the algorithm identified 84.77% of clinically diagnosed cases (*ICD-10* J69.0) and revealed 1891 additional cases undetected by clinical practice, representing a 62.6% increase in detection compared with standard approaches [40].

Patients with R13 had a mean stay of 15.5 (SD 19.7) days, similar to one of our studies with an overall hospital stay of 14.0 (SD 11.2 days) [38]. The prevalence of AP was 10.7%, lower than in another study with 16.3% [41]. That could also be due to the underdiagnosis of AP and the lack of standardized clinical criteria for its diagnosis [42]. In the case of MN, the prevalence was 4.2%, much lower than that reported in several of our studies, which were 50.6%, 45.5%, and 45.9% in patients hospitalized with COVID-19 disease [5,38,39]. The prevalence of DH was 3.8%, also lower than that reported in one of our studies with a prevalence of 19%‐100% [43]. Again, MN and DH are seldom evaluated in older patients, and this underdiagnosis was even more severe during the COVID-19 pandemic, clearly evidenced in this study. Of the R13-OD patients, 15.0% (1762/257,541) were institutionalized upon discharge, a lower percentage compared with 2 of our previous studies with 27.5% and 15.2% [5,37]. This could be due to different political management as, during the first wave, improvised discriminatory decisions were made regarding the admission and destination of discharge in these older patients [44]. The mortality rate for R13-OD was 8.7% (1207/257,541) in all patients and increased to 11.8% (956/104,898) in patients aged 70 years or older. Other studies have shown mortality prevalence similar to ours, of 11.4% during the first wave in Spain [45] and 11.0% in China [46]. On the other hand, patients with H-AIMS-OD had a higher prevalence of ICU admission than R13-OD patients in our study. Hospitalized patients in the 3 groups (R13-OD, H-AIMS-OD, and with no R13-OD and L-AIMS-OD) had a higher prevalence of complications associated with OD and higher total mortality than patients treated in primary care. This is because patients with the worst prognosis were hospitalized. Prevalences of complications in hospitalized patients were 24.2% for AP, higher than that reported in one of our studies (16.3%) [41]. A prevalence of 10.6% in MN was significantly lower than the prevalences reported in several studies (50.6%, 45.5%, and 45.9%) with patients with COVID-19 disease [5,37,47]. The prevalence of DH was 8.0%, which is lower than the prevalence reported in our previous studies (19%‐100%) [43].

We found that neuroleptic use, cerebral infarction, and being older than 70 years were independent risk factors for OD diagnosis according to both R13-OD and H-AIMS-OD in the overall population. These results are consistent with our previous publications on the pathophysiology of OD in older patients [9,30,48]. In another study [38], similar risk factors were shown, such as associated complications and the intake of drugs such as anticholinergics, muscle relaxants, and opioids. In the case of neuroleptic use, the study by Miarons et al [49,50] showed that both typical and atypical antipsychotics can be associated with OD. For hospitalized patients, the main risk factors for OD diagnosis according to *ICD-10* were dementia, being aged 70 years and older, and delirium. These risk factors are consistent with the results obtained in one of our studies of 255 hospitalized patients with dementia, where the risk factors for OD were older age, poorer functioning, poorer nutritional status, and greater severity of dementia [51]. On the other hand, being aged 70 years and older and chronic obstructive pulmonary disease were risk factors for patients with H-AIMS-OD. In a systematic review on swallowing function and chronic respiratory diseases, results indicated that chronic respiratory diseases increase the prevalence of OD [52]. Other risk factors for OD according to R13-OD were hospital stay, hospital admissions, and comorbidities assessed with the Charlson index. In contrast, for OD with H-AIMS-OD, these previous factors, including ICU admission, became protective. This could be explained because, due to the excess demand during the pandemic, younger patients and those with fewer comorbidities were given priority to ICU admission (for critical patients) as they had a better chance of survival [5,38,47]. Importantly, our population-level findings derived from routine clinical data show strong external consistency with recent prospectively collected clinical cohorts. In this regard, the risk factors we have identified are in line with those reported in patients with COVID-19 disease. Zayed et al [12] observed that advanced age, longer duration of COVID-19 symptoms, presence of ageusia and anosmia, dysphonia, ICU admission, lower oxygen saturation, and use of mechanical ventilation were significantly associated with dysphagia in 500 hospitalized patients [12]. Their subsequent instrumental study, using FEES, confirmed the persistence of swallowing abnormalities in patients with post-COVID-19 disease, including a delayed swallowing reflex and altered laryngeal sensitivity [13].

The length of hospital stay, the number of admissions, and a high Charlson index emerged as relevant risk markers, particularly among older patients, reflecting their increased vulnerability and more severe clinical prognosis. In the H-AIMS-OD group, a broader age distribution was observed. In this context, variables that traditionally represent risk factors in older populations appear as protective factors for H-AIMS-OD, largely due to the management strategies applied during the COVID-19 pandemic [53], which prioritized younger patients in decisions regarding hospitalization, length of stay, and ICU admission. Consequently, these variables become statistically associated with younger age and are therefore perceived as protective in this subgroup.

Finally, in primary care patients, the only risk variable found was being aged 70 years and older. Age has been previously determined in many studies as a risk factor for dysphagia [9,46,54-56]. In addition, aging is one of the main causes of the disease [56] and in the community (primary care level), OD has a high prevalence (27%) despite the lower complexity of patients in that setting [57].

The main result of our study is the underdiagnosis of OD in patients with COVID-19 disease, since we know that approximately only 20% (50/253) of patients with OD were correctly diagnosed. The clinical and instrumental diagnosis of OD during COVID-19 disease was hampered by the danger of contagion and overstretched health care workers, but AIMS-OD provides systematic and universal screening for OD during hospital admission, allowing the most appropriate diagnostic and therapeutic strategies to be selected for each patient. This is a big step forward and represents a marked improvement in health care: patients with OD who are hospitalized or discharged due to COVID-19 disease have the right to be diagnosed and treated for OD, their family and caregiver organizations gain a deeper understanding of OD, health care professionals can identify and effectively manage high-risk (AIMS-OD>0.5) patients, and health care providers improve their results and lower costs. Currently, there are 2 more services being developed, AIMS-AP for AP and AIMS-MN for MN. AIMS-AP is in the in vitro development phase and has a database of more than 5000 patients and good psychometrics to detect patients with AP. In parallel, awareness of OD and its complications should be increased in all health care settings through education and promotion of evidence-based clinical practice.

Our findings are consistent with a number of studies that use large-scale population-based epidemiological methodologies to examine changes in disease prevalence before and during the COVID-19 pandemic. A recent ecological study analyzing more than 103.000 patients in South Korea showed that the incidence of Bell palsy decreased significantly during the nationwide vaccination and infection phases of the pandemic compared with the pre-COVID-19 baseline, highlighting how pandemic-related public health interventions may have influenced disease patterns [58]. Similarly, an analysis of national trends examining asthma prevalence using data from more than 206,000 participants found that while asthma prevalence increased in the prepandemic era, it decreased significantly during the pandemic period, suggesting that factors such as reduced air pollution, universal mask wearing, and social distancing may have contributed to these changes [59]. These studies, like ours, demonstrate the value of leveraging national health databases to identify underdiagnosed conditions and monitor changes in disease prevalence during public health crises. These studies highlight the potential of AI-based screening tools, such as AIMS-OD, not only to address current underdiagnosis but also to serve as scalable screening systems for emerging health conditions in future pandemics.

### Limitations

This study has some limitations. First, AIMS-OD was not validated in patients with COVID-19 disease; however, this limitation reflects the extraordinary clinical context of the pandemic, during which instrumental and bedside swallowing assessments were often not feasible. Second, the database had some missing values and the absence of important variables for OD such as Barthel Index, among others, could have affected our ability to better estimate the risk with AIMS-OD of the patients studied and their risk factors. This has meant that the calculation of true prevalence was affected in the estimation of the reality of OD.

This study shows a high prevalence of patients with COVID-19 disease at high risk of OD during the first wave of the pandemic in Catalonia and that most of them (53,832/65,576, 82.1%) were underdiagnosed and thus, R13-OD–detected patients were very few. In contrast, massive screening with AIMS-OD allowed us to identify a large group of patients with COVID-19 disease with a high risk of OD and associated complications and poor clinical outcomes aligned with those of R13-OD patients. We believe that systematic screening with AIMS-OD will aid clinical decision-making and improve the diagnosis of OD, still a neglected condition in several phenotypes of patients, and especially in the context of a pandemic.

Risk factors for OD in the whole population included neuroleptic use, cerebral infarction, and age. Hospitalized patients had additional risk factors such as delirium and dementia. On the other hand, primary care patients had only 1 risk factor, age. Our findings further suggest that OD is a significant problem in patients with COVID-19 disease. Early identification of patients at risk of OD using tools such as the AIMS-OD will help improve the management and clinical outcomes of these patients.

### Conclusions

Our findings further suggest that OD is a significant and underrecognized problem in patients with COVID-19 disease, especially in those of advanced age and with comorbidities. The high proportion of underdiagnosed cases observed in this study highlights the limitations of routine clinical detection during the pandemic. Systematic screening using tools such as AIMS-OD may facilitate earlier identification of patients at risk, support clinical decision-making, and improve the management and clinical outcomes of these patients.

## Abbreviations

AI: artificial intelligence
AIMS-OD: Artificial Intelligence Massive Screening for Oropharyngeal Dysphagia
AP: aspiration pneumonia
API: application programming interface
DH: dehydration
EHR: electronic health record
ES: expert system
FEES: fiberoptic endoscopic evaluation of swallowing
H-AIMS-OD: high-risk AIMS-OD
*ICD*: *International Classification of Diseases*
*ICD-10*: *International Classification of Diseases, Tenth Revision*
*ICD-11*: *International Classification of Diseases, 11th Revision*
*ICD-9*: *International Classification of Diseases, Ninth Revision*
ICU: intensive care unit
L-AIMS-OD: low AIMS-OD risk
ML: machine learning
MN: malnutrition
OD: oropharyngeal dysphagia
PADRIS: *Program of Dades per a la Recerca i la Innovació en Salut*
